# Immunotherapy for Esophageal Squamous Cell Carcinoma

**DOI:** 10.1007/s11912-017-0590-9

**Published:** 2017-03-30

**Authors:** Takashi Kojima, Toshihiko Doi

**Affiliations:** Department of Gastroenterology and Gastrointestinal Oncology, National Cancer Center Hospital East, 6-5-1, Kashiwanoha, Kashiwa, Chiba 277-8577 Japan

**Keywords:** Esophageal neoplasms/TH, Immunotherapy

## Abstract

Esophageal squamous cell carcinoma have been frustrating to treat, with slow progress made on extending survival. Immunotherapy targeting immune checkpoints, T cells, and infiltrating lymphocytes has shown promise in early studies. The efficacy of pembrolizumab and nivolumab is encouraging. Anti-chemokine receptors and oncolytic viruses are also making headway against these stubborn tumors; improved results when immune checkpoint inhibitors are combined with radiation therapy are eagerly anticipated. Adoptive T cell therapy and vaccines are also under development. The importance of a multidisciplinary approach cannot be emphasized enough.

## Introduction

The two histological subtypes of esophageal/esophagogastric junction (EGJ) carcinoma, adenocarcinoma (ADC) and squamous cell carcinoma (SCC), have different risk factors and clinicopathological features. Although not all clinical trials have broken down their results according to subtype, the differences in genomic alterations in biologic pathways between ADC and SCC are beginning to be elucidated [1, 2]. Therapies targeting HER2 (trastuzumab) and vascular endothelial growth factor (ramucirumab) are applicable only to adenocarcinomas [3–5] of the esophagus and EGJ.

Esophageal cancer is ranked as the sixth leading cause of cancer-related deaths. In 2012, 400,000 deaths were reported globally due to esophageal cancer [6]. Although the incidence of adenocarcinoma is increasing in Western countries, esophageal squamous cell carcinoma (ESCC) is the major histology in Asian countries including Japan.

Smoking and alcohol intake, known to be the major risk factors for this cancer, exert synergistic effects on carcinogenesis [7]. Long-term exposure to carcinogens related to smoking and aldehyde, a metabolite of alcohol, produces DNA damage and a range of genetic changes [8]. Although driver gene mutations have not been detected in ESCC, the somatic mutation rate in ESCC is relatively high compared with other solid tumors [9, 10].

A multidisciplinary approach, including surgery and radiotherapy, is important for ESCC. Chemotherapy is standard for patients with distant metastases. Commonly used agents include 5-fluorouracil, platinum agents, and taxanes, though they are associated with limited clinical benefit [11–14].

Although molecular targeting agents remarkably improve the outcome of several types of solid tumors, no agents have shown clear efficacy in ESCC up to now [15, 16]. However, the pace of development of cancer immunotherapies is accelerating. Clinical evidence of the efficacy of immune checkpoint inhibitors and adoptive immunotherapies, complete with tumor-infiltrating lymphocytes and tumor-specific receptor gene-modified T cells, herald the onset of a new era in cancer immunotherapy.

This review summarizes the most recent status of immunotherapy for ESCC (Table 1).

**Table 1:** List of trials with immune checkpoint therapies for ESCC or solid tumors including ESCC

	Target	Agent	Sponsor	Line	Phase	Number		Comment
Checkpoint inhibitors	PD-1	ONO-4538	ONO/Bristol-Myers	2nd	ΙΙΙ	390	NCT02544737	
		ONO-4538	Bristol-Myers	Adj.	ΙΙΙ	760	NCT02743494	
		MK-3475	MSD	2nd	ΙΙΙ	600	NCT02564263	
		MK-3475	MSD	3rd	ΙΙ	100	NCT02559687	
		MK-3475	MSD	–	ΙΙ	14	NCT02830594	Palliative CRT
		MK-3475	Washington University School of Medicine	1st	0	15	NCT02642809	Palliative CRT
	PD-L1	MEDI-4736	Ludwig Institute for Cancer Research	1st	Ι/ΙΙ	75	NCT027352239	Definitive CRT
		MEDI-4736	Samsung Medical Center	Adj.	rΙΙ	84	NCT02520453	
Peptide vaccine	NY-ESO-1	IMF-001	Mie University	Adj.	rΙΙ	70	UMIN000007905	
		S-588410	Shionogi & Co., Ltd.	Adj.	ΙΙΙ	270	UMIN000016954	
	HSP105	HSP105-derived peptide vaccine	National Cancer Center Hospital East	Salvage	Ι	15	UMIN000017809	
Adoptive T cell therapy	MAGE-A4	TBI1201	Mie University	Salvage	Ι	12	NCT02096614	
	NY ESO-1	TBI-1301	Mie University	Salvage	Ι	12	NCT02366546	
		TBI-1301	University Health Network, Toronto	Salvage	Ι	15	NCT02869217	
		Anti-NY ESO-1 TCR-transduced T cells	Shenzhen Second People’s Hospital	Salvage	Ι	36	NCT02457650	
Oncolytic viruses		OBP-301	Okayama University	1st	Ι	12	UMIN000010158	Definitive CRT
Combination cancer immunotherapy	PD-L1/CTLA4	MEDI4736/tremelimumab	Ludwig Institute for Cancer Research	1st	Ι/ΙΙ	75	NCT02735239	
	PD-1/CCR4	ONO-4538/KW-0761	Kyowa Hakko Kirin Company, Limited	Salvage	Ι	108	NCT02476123	
		ONO-4538/KW-0761	Osaka University	Neoadj.	Ι	18	UMIN000021480	

## Checkpoint Inhibitors

Immune checkpoints are downregulators of the immune response. Immune checkpoint blockade has drastically changed the treatment of melanoma, and its effectiveness is being explored in other tumor types, including gastrointestinal malignancies [17]. Programmed death-ligand 1 (PD-L1) expression rates, associated with favorable overall survival (OS) in ESCC, have been reported to range from 41.9 to 84.5% [18–20]. Since PD-L1 expression is known to be induced by activated T cells, agents targeting PD-L1 may be effective in PD-L1+ ESCC patients [21].

An increase in the burden of nonsynonymous mutations in tumors has been associated with improvements in objective response and long-lasting clinical benefit as well as progression-free survival (PFS). Efficacy has also been correlated with molecular smoking signature, a higher neoantigen burden, and mutations in the DNA repair pathway. Each of these factors has also been associated with mutation burden [22].

As mentioned above, the somatic mutation rate in ESCC is relatively high compared with that of other solid tumors [9, 10]. Patients with esophageal cancer sometimes develop head and neck cancer at the same time or after treatment, because these cancers can arise by the same etiology and carcinogenesis. This possibility was suggested by the “field cancerization theory,” first proposed in 1958, as due to exposure of multiple portions of the upper digestive tract to the same carcinogen, such as tobacco or alcohol [23].

Pembrolizumab, an anti-PD-1 antibody, received FDA approval for use in patients with recurrent or metastatic head and neck squamous cell carcinoma and disease progression concurrent with or following platinum-containing chemotherapy. This approval was based on data obtained from the KEYNOTE-012 study; this study included subjects with an ECOG performance score of 0 or 1 who had recurrent or metastatic head and neck squamous cell carcinoma and disease progression at the time of or subsequent to platinum-containing chemotherapy or after platinum-containing chemotherapy given as part of induction, concurrent, or adjuvant therapy. According to the FDA approval summary, the objective response rate for these 174 patients was 16%, with a response lasting ≥6 months in 23 of 28 responding patients [24].

Doi et al. showed that pembrolizumab was active in pretreated esophageal cancer patients with PD-L1-expressing tumors (>1% PD-L1-positive tumor cells and/or tumor stroma), with a partial response (PR) rate of 30.4% (40.0% for adenocarcinoma, 29.4% for squamous cell); there were no complete responses (CR). A total of 52.2% of patients showed some degree of tumor shrinkage and manageable side effects [25••]. In this study, the six-gene signature (*IDO1*, *CXCL10*, *CXCL9*, *HLA-DRA*, *STAT1*, *INF-*γ) captures part of a complex signaling pathway related to pre-existing INF-γ adaptive immune response within the tumor microenvironment [26••]. Kojima et al. also reported data on nivolumab, a human monoclonal IgG4 PD-1 immune checkpoint inhibitor antibody, for patients with advanced ESCC not preselected by PD-L1 status, with a PR rate of 15.6% and a CR rate of 1.6%. The median overall survival (OS) was 12.1 months in 64 evaluable patients. The incidence of drug-related serious adverse events was 11 events (9/65 patients): lung infection (2 [3.1%]), dehydration (2 [3.1%]), interstitial lung disease (2 [3.1%]), diarrhea (1 [1.5%]), fatigue (1 [1.5%]), hepatic dysfunction (1 [1.5%]), hypernatremia (1 [1.5%]), and dyspnea (1 [1.5%]). The patients with grade 3 pneumonitis improved with supportive care. There were no treatment-related deaths [27•].

The results of these studies show that anti-PD-1 antibody therapy is a potential treatment in patients with ESCC. Other studies of interest include a phase III trial of nivolumab (Opdivo®, Squibb, Princeton NJ, USA), an anti-PD-1 antibody, for patients with unresectable advanced or recurrent esophageal cancers (JapicCTI-153026; NCT02569242). A phase III study of pembrolizumab (Keytruda®, Merck, Kenilworth, NJ, USA) is being conducted on patients with advanced ESCC or ADC in the esophagus or esophagogastric junction progressing after the first-line therapy (NCT02564263). Other anti-PD-1 antibodies are being evaluated for adjuvant therapy.

Chemokines and chemokine receptors control the migration and homing of cells in the body. One promising target for novel cancer immunotherapy is the CC chemokine receptor 4 (CCR4). Mogamulizumab (KW0761), the first biologic agent targeting CCR4, obtained Japanese licensure for use in the treatment of adult T cell leukemia/lymphoma (ATL) in 2012. CCR4 is expressed on CD45RA–FOXP3 high CD4^+^ effector regulatory T (Treg) cells. In a phase Ia study in patients with CCR4-negative advanced or recurrent solid cancers, mogamulizumab was tested for its ability to deplete effector Treg cells and thereby strengthen the anti-cancer immune response. The tolerability and safety of mogamulizumab by infusion at a dose between 0.1 and 1.0 mg/kg were demonstrated, and dose-limiting toxicity was not observed. Four of the ten patients (three of seven lung and one of three esophageal cancer patients) showed stable disease throughout treatment and were long-term survivors [28•, 29]. Now, a phase Ib study in 40 patients with advanced or recurrent cancer who are assigned at a 1:1 ratio to 0.1 or 1.0 mg/kg mogamulizumab is ongoing (NCT01929486).

Checkpoint inhibition alone is not enough to promote tumor regression in a majority of cancer patients. For a robust therapeutic immune response, it is important not only to block negative regulatory receptors but also to enhance and trigger positive costimulatory signals. Novel pharmacologic agents that target other immune checkpoints and costimulatory receptors, including CTLA4, OX40, 4-1BB, and LAG-3, are currently being evaluated in a wide variety of tumors, including ESCC. In addition, there are significant efforts underway to evaluate combinations of immune checkpoint inhibitors with a variety of other anti-cancer therapies, including radiation, surgery, and cytotoxic chemotherapy.

## Combining Immune Checkpoint Inhibitors with Radiation

Recently, increased knowledge of T cell regulatory mechanisms has resulted in the development of a novel class of immune checkpoint inhibitors with strong clinical efficacy against a wide range of malignancies, including those which were not previously considered sensitive to immunotherapy. This has led to a renewed focus on the possibility of synergy with older anti-cancer therapies such as radiation therapy (RT).

The use of RT as definitive or palliative treatment for some malignancies has been well established. Recently, however, the possibility of using RT in combination with immunotherapies has gained attention. In addition to control of tumor growth, RT exerts a range of immunomodulatory effects on the tumor and its microenvironment. These serve to prime the tumor for an immune-mediated response [30].

Radiation is the foundation of treatment for ESCC. Approaches to integrate immune checkpoint inhibition into radiation-based therapy bring a range of challenges. Radiation is known to produce immune-mediated abscopal effects; proposed mechanisms include the recruitment of T cells into the microenvironment, secretion of cytokines, and enhancement of tumor antigen presentation [31, 32]. Interestingly, pre-clinical data indicate that increased PD-L1 expression in irradiated tumors suppresses the radiation-induced anti-tumor properties of effector T cells [33]. This has many therapeutic implications, particularly in locally advanced ESCC, in which the anti-tumor response might by maximized by the combination of anti-PD-1 antibodies and radiation. Radiation increases tumor antigen presentation and potentiates the checkpoint inhibitor-induced anti-tumor response [34]. A number of clinical trials to evaluate pembrolizumab in combination with radiation are currently underway. Two studies include the following:

- Pembrolizumab with brachytherapy for the initial treatment of metastatic esophageal cancers (NCT02642809).
- Pembrolizumab and palliative radiation therapy for patients with metastatic esophageal, stomach, or EGJ cancer (NCT02830594).

## Peptide Vaccines

One area of oncology research attracting substantial and growing interest is active cancer immunotherapy. One attractive option is peptide vaccines, which involve the use of isolated immunogenic tumor-associated antigen (TAA) epitopes to cause an anti-cancer immune response. Peptide vaccines are easily administered and are minimally toxic. Multiple TAA-derived peptides have been identified and evaluated with various vaccine strategies currently under clinical investigation.

Findings to date have suggested that the use of vaccines in patients with minimal residual disease may be more efficacious than targeting patients with broadly disseminated metastatic disease that tends to confer immunosuppression.

Clinical trials have indicated that achieving maximum efficacy may require that vaccines be tailored for particular cancer subtypes. Further, many of the large number of immunomodulators now in development have shown possible synergy with peptide vaccines. One current focus of attention is tumor-specific neoantigens, and clinical trials of individualized peptide vaccinations that target individual neoantigens are now in progress.

Progressively growing tumors contain mutant tumor antigen-specific T cells. These are reactivated after treatment with anti-PD-1 and/or anti-CTLA-4, and although they show some overlap, their transcriptional profiles are mostly treatment-specific, giving them the ability to mediate tumor rejection. Tumor-specific mutant antigens are thus not only major targets in checkpoint blockade therapy but can be further used in the development of personalized and cancer-specific vaccines as well as in the investigation of the mechanisms underlying the various checkpoint blockade treatments [35].

One peptide, the 20-mer NY-ESO-1f, contains many epitopes that are recognized with distinct specificity by CD4 and CD8 T cells. Of the ten patients, two vaccinated patients with lung cancer and one with esophageal cancer showed stable disease. This study shows that the NY-ESO-1f peptide vaccine was well tolerated and elicited humoral, CD4, and CD8 T cell responses in immunized patients [36]. The immune response induced by the vaccination could make the prognosis better for advanced ESCC.

In a phase I dose-escalation trial of two dose levels of CHP-NY-ESO-1 vaccine, complex vaccine (drug code: IMF-001) consisting of recombinant NY-ESO-1 protein and cholesteryl hydrophobized pullulan (CHP), in patients with ESCC [37], the CHP-NY-ESO-1 vaccine was confirmed to be safe and to induce immunogenicity in patients with antigen-expressing ESCC. The higher dose induced an antigen-specific immune response and better survival benefits, even for patients with a poorer prognosis. From the results of these studies, a randomized multicenter phase II trial of adjuvant IMF-001 after curative resection for esophageal cancer with NY-ESO-1 antigen is now ongoing (UMIN000007905).

The safety of combination treatment using a multipeptide vaccine with CRT has been confirmed, and its use may be effective in patients with unresectable ESCC [38].

However, peptide vaccine therapy targeting common self-antigens has not yet been approved in Japan. Some of these vaccines have been investigated in ESCC patients.

Ongoing trials are as follows:

- A multicenter, randomized, double-blind, placebo-controlled, phase III study of S-588410, a cancer peptide vaccine containing five human leukocyte antigen (HLA)-A*2402-restricted epitope peptides, as adjuvant therapy after curative resection in patients with esophageal cancer (UMIN000016954).
- A randomized multicenter trial of adjuvant IMF-001 with NY-ESO-1 antigen (phase II study) after curative resection for esophageal cancer (UMIN000007905).
- Phase 1 study of HSP105-derived peptide vaccine for patients with advanced esophageal cancer/colorectal cancer (UMIN000017809).

## Adoptive T Cell Therapy

Another promising treatment is adoptive immunotherapy. This involves the administration of T cells which have been manipulated to target a tumor. These therapies have shown marked efficacy in the treatment of hematologic malignancies as well as certain solid tumors, including melanoma.

Genetic tools which efficiently engineer T cell specificity and enhance T cell function have been developed. Chimeric antigen receptors (CAR) use antibody-variable segments to target specificity at cell surface molecules. T cell receptors (TCR) are used to redirect T cells to intracellular target proteins; these are present within the peptide-binding groove of HLA molecules in fragment form.

A clinical trial of CAR-modified T cells redirected to target the B cell lineage antigen CD19 produced marked clinical results in patients with chronic lymphocytic leukemia [39]. Similar impressive responses were obtained in patients with melanoma and synovial cell carcinoma using TCR-modified T cells redirected to target MART-1, a melanocyte lineage antigen, and NY-ESO-1, a testis-cancer antigen [40]. Nevertheless, most of these clinical responses were associated with both on- and off-target toxicity, and death from complications has occurred in some patients receiving gene-modified T cells.

In a phase I trial which evaluated the use of TCR gene-transduced T cell transfer in recurrent MAGE-A4-expressing ESCC, TCR-transduced cells were identified in the peripheral blood for 1 month in dose-proportional levels. In five patients, these persisted for more than 5 months. Despite this long persistence of the transferred T cells, seven patients showed tumor progression within 2 months after treatment, whereas three patients with minimal baseline tumor lesions survived longer than 27 months [41•].

Because they allow for more precise T cell targeting, the emerging technology of chimeric antigen receptor T cells (CAR-T) has transformed the field of adoptive T cell therapy. A number of trials are now investigating adoptive T cell transfer techniques in patients with ESCC. These include the following:

- A phase I study of T cells engineered to recognize the MAGE-A4 marker (TBI-1201) in patients with solid tumors, including ESCC (NCT02096614).
- A phase I study of T cells engineered to recognize the NY-ESO-1 marker (TBI-1301) in patients with solid tumors, including ESCC (NCT02366546).
- T cell receptor-transduced T cell targeting NY-ESO-1 for treatment of patients with NY-ESO-1-expressing malignancies (NCT02457650).

## Oncolytic Viruses as Immunotherapy

The use of oncolytic viruses for previously untreatable cancers has recently been proposed [42]. These viruses selectively replicate in tumor cells, causing them to lyse, and their use as novel anti-cancer agents has been closely investigated. These vectors are assumed to undergo selective viral propagation within the tumor cell and cause virus-mediated lysis of tumor cells, while having no effect on normal tissues.

Talimogene laherparepvec (T-VEC), the first oncolytic virus to show positive results in a phase III clinical trial, is derived from the herpes simplex virus [43]. It received FDA approval for advanced metastatic melanoma in October 2015.

Engineered oncolytic viruses induce tumor cell lysis and inflammation. They increase T cell numbers and activity and thereby strengthen the anti-tumor response after treatment with anti-CTLA-4 or anti-PD-1 agents [44]. These combinations might enhance efficacy by negating the ability of the local tumor microenvironment to suppress the induced immune response [45]. Some reports have indicated that combination of talimogene laherparepvec with a checkpoint inhibitor might show promising anti-tumor activity [46, 47]. Future research should be directed at how better to suppress anti-viral immune responses and minimize pathology while simultaneously enhancing anti-tumor immunity.

A recent study described a phase I/II trial of a novel telomerase-specific oncolytic virus, OBP-301, given by endoscopic intratumoral administration together with radiation in elderly patients with ESCC. This study enrolled six patients in a single cohort. Objective responses were CR in two patients and partial response (PR) with tumor regression in one [48•]. The results showed the feasibility and tolerability of multiple courses of endoscopic OBP-301 injection together with locoregional radiotherapy in patients with ESCC as well as apparent clinical benefit. Endoscopic treatment for ESCC, such as endoscopic mucosal resection, is actively performed worldwide; endoscopic intratumoral injection of oncolytic virus is not a difficult technique. These findings indicate the potential of oncolytic virotherapy in the treatment of ESCC. A clinical study of a tumor-selective oncolytic adenovirus (Telomelysin) in combination with ionizing radiation for head and neck and thoracic malignant tumors, including esophageal cancer (UMIN000010158), is ongoing.

## Combination Cancer Immunotherapy

Immune responses against tumors involve positive and negative feedback loops of numerous host immune cells and molecules around the tumor. Combination cancer immunotherapy, using therapies with different modes of action has attracted much interest and is now considered a mainstream developmental approach. Combination therapies include conventional chemotherapy as well as biological agents and radiotherapy. Any future success is likely contingent on the ability to maximize the gathering of specific information on the interaction of immunotherapies with concomitant medications with regard to their mechanisms of action, doses, and dosing regimens.

CTLA-4 and PD-1 regulate different inhibitory pathways, and combination therapies with antibodies targeting both molecules have been shown to improve anti-tumor response in a mouse model. In a phase I clinical trial of anti-CTLA-4 blockade in combination with anti-PD-1 inhibition, the combination of ipilimumab and nivolumab provided high tumor regression rates in patients with melanoma [49]. In fact, the results of the randomized phase III study evaluating this combination in melanoma patients have been recently communicated, which in 2015 led to FDA approval. These promising results have led to a number of phase I trials currently testing combination treatment with an anti-CTLA-4 and an anti-PD-1/PD-L1 antibody.

Ongoing trials are as follows:

- Study of combination therapy with mogamulizumab (KW-0761) and nivolumab (ONO-4538/BMS-936558) in subjects with advanced solid tumors, including esophageal cancer (NCT02476123).
- Phase I study of pre-operative combination therapy with mogamulizumab (anti-CCR4) and nivolumab (anti-PD-1) against solid cancer patients (UMIN000021480).

## Conclusion

Immunotherapy for the treatment of patients with advanced ESCC has shown significant recent advances.

In particular, the value of immune checkpoint inhibitors has been comprehensively demonstrated in the treatment of melanoma and nonsmall cell lung cancer, and emerging efficacy data in advanced ESCC. Among new approaches, therapeutic peptide vaccines, oncolytic Viruses, and adoptive T cell therapy have shown potential in treatment of ESCC. These are under active development. The complex biology and variety of tumor evasion strategies means that combination strategies are very important for future development.
